# Editorial: The intersection of cognitive, motor, and sensory processing in aging: Links to functional outcomes, Volume I

**DOI:** 10.3389/fnagi.2022.1009532

**Published:** 2022-08-30

**Authors:** Jennifer L. Campos, Uros Marusic, Jeannette R. Mahoney

**Affiliations:** ^1^KITE – Toronto Rehabilitation Institute, University Health Network, Toronto, ON, Canada; ^2^Department of Psychology, University of Toronto, Toronto, ON, Canada; ^3^Institute for Kinesiology Research, Science and Research Centre Koper, Koper, Slovenia; ^4^Department of Health Sciences, Alma Mater Europaea—ECM, Maribor, Slovenia; ^5^Division of Cognitive and Motor Aging, Albert Einstein College of Medicine, Bronx, NY, United States

**Keywords:** sensory performance, motor performance, cognitive performance, multisensory integration (MSI), aging

Integral to the ability to carry out everyday tasks is the successful coordination of cognitive, motor, and sensory processing in the brain. However, age-related changes alter cognitive, motor, and sensory functioning, as well as their interactions, which can affect daily functioning in older adults. Investigations of the interplay across these three systems has been somewhat limited in the aging literature, though research examining relationships across various combinations (e.g., cognitive & motor, motor & sensory, sensory & cognitive, and multisensory) has been plentiful.

For instance, there is extensive research on the link between unisensory impairments and motor decline in aging (Center for Disease Control, 2010). That is, unisensory impairments have been linked to slower gait speed (Kaye et al., 1994), worse functional decline (Laforge et al., 1992), increased risks of falls (Lord and Ward, 1994; Judge et al., 1995; Camicioli et al., 1997; Lord et al., 1999) and poorer quality of life (Carabellese et al., 1993). Age-related hearing loss has also been associated with a greater risk of mobility problems, including a three-fold increased risk of falls (Lin and Ferrucci, 2012; Jiam et al., 2016; Campos et al., 2018). Impairments in balance have also been associated with inefficient interactions between musculoskeletal and sensory systems (Shumway-Cook and Woollacott, 2012), which are often compromised in aging (Lord et al., 2007). Furthermore, deficits in multisensory integration processes, specifically visual-somatosensory and auditory-visual interactions, have been associated with impairments in gait (Mahoney and Verghese, 2018), balance (Mahoney et al., 2014, 2019), falls (Setti et al., 2011; Lupo and Barnett-Cowan, 2018; Mahoney et al., 2019) and individuals with hearing loss have poorer vestibular functioning (Gabriel et al., 2022).

Mobility declines are common in aging, especially in older adults with mild cognitive impairment and dementia (Beauchet et al., 2008; Verghese et al., 2008). The association of executive functioning with balance (Woollacott and Shumway-Cook, 2002; Zettel-Watson et al., 2017), gait (Verghese et al., 2007, 2008; Holtzer et al., 2012), and falls (Hausdorff and Yogev, 2006; Holtzer et al., 2007) in aging is well-established. The link between cognitive and motor function in aging reveals that the prefrontal cortex plays a critical role in successful gait and cognition (Beauchet et al., 2016). In fact, evidence of combined slow gait and subjective cognitive complaints are indicators for Motoric Cognitive Risk syndrome [MCR]—a pre-dementia syndrome first proposed by Verghese et al. (2013, 2014) almost a decade ago.

Age-related sensory loss is also associated with cognitive decline and dementia (Baltes and Lindenberger, 1997; Albers et al., 2015; Livingston et al., 2017, 2020; Lin, 2011). For example, hearing loss has been identified as the top potentially-modifiable risk factor for dementia (Livingston et al., 2017, 2020). Links between impairments in multisensory integration and cognition have also been observed, and highlight the need for increased development of clinical translational multisensory integration investigations (Mahoney and Barnett-Cowan, 2019) and tools. Multisensory integration has been associated with attention-based performance (Poliakoff et al., 2006; Hugenschmidt et al., 2009; Mahoney et al., 2012; Mahoney and Verghese, 2020) and individuals with mild cognitive impairment demonstrate differences in multisensory integration compared to those with normal cognition (Chan et al., 2015). Further, the mediating effect of cognitive impairment on the association between poor visual-somatosensory integration and slow gait, as well as worse balance, has recently been documented (Mahoney and Verghese, 2020) and investigations establishing visual-somatosensory integration as a novel marker for Alzheimer's disease are currently underway.

Greater understanding of the neural connections between and across (multi)sensory, motor, and cognitive processing in both healthy and pathological aging is clearly warranted. The express purpose of this compilation is to collectively consider the existing inter-relationships between sensory, motor, and cognitive functioning, as well as to promote novel lines of research examining the intersection of these systems in aging. For this special issue, it was expected that contributions would consider age-related functionality of more than one system (e.g., sensory, motor, and/or cognitive) and the implications of these systematic interactions on important functional outcomes, including but not limited to clinical, motor, and social outcomes to name a few. Gaining further knowledge about the successful (or unsuccessful) intersection of these systems could prove valuable for older adults, especially with regard to refining and restructuring multimodal interventions aimed at preventing declines, reducing disability, and maintaining functional independence, in an effort to enhance quality of life.

In terms of sensory and motor interactions, Marusic, Peskar, et al. set out to determine the root cause of age-related sensorimotor slowing using electrophysiological measures and a simple visual reaction time test. Their results reveal that age-related slowing of sensorimotor activity originating in motor cortex is relatively uninfluenced by early-visual stimulus processing but related to later-visual processes linked to higher-order cognitive function like attention and working memory. In a study examining the effectiveness of multisensory training for improving self-motion perception, Gabriel et al. *(this issue)* found that visual-vestibular training improves visual heading perception, particularly in older adults. Results reveal the potential beneficial effects of multisensory training in aging and provide clues that multisensory training could enhance future rehabilitation programs that target age-related mobility declines. Scurry et al. present preliminary support of drastic reductions in multisensory integration (*via* top-down inhibitory control mechanisms) in older adults with a history of falling. In addition, Wunderlich et al. define a Mobile Brain/Body Imaging (MoBI) approach to gain deeper insights into the neural dynamics underlying cognitive and motor interactions during several dual-task conditions in young and old adults with hearing impairment.

In a novel dual-task paradigm, Ward et al. demonstrates cognitive-motor dual-task interference effects on balance (i.e., postural control) irrespective of age and cognitive task load. In an exercise study, Tsai et al. investigated the acute effect of high-intensity interval *vs*. moderate-intensity continuous exercise on executive-related oculomotor performance in aging and found that high-intensity interval exercise appears to be more effective in terms of modulating oculomotor control. Li N. et al. examined the effect of age on visuomotor adaptation and found that compared to the younger adults, older adults had less adaptation to visual feedback perturbation in a reaching task. However, the authors further that while the effect of aging on visuomotor adaptation was not associated with chronological age, it was correlated with the declines in cognitive performance. Di Tella et al. examined functional and structural neural changes in old vs. young adults and found reduced cortical thickness of the mirror neuron system, coupled with increased activation in premotor and prefrontal areas indicative of age-related changes in both cognitive and motor neural systems.

Using sophisticated structural equation modeling, Xue et al. reveal that cognitive function significantly interacts with frailty and urge that both should be considered when developing tailored interventions so as to further improve overall health outcomes and quality of life for older adults. Marusic, Verghese et al. examined the effect of cognitive training using computerized brain games on mobility measures in healthy, active older adults and found that cognitive training had both near- (executive function & processing speed enhancements) and far- (dual-task gait speed enhancement) transfer effects concurrently related to increased activation over sensorimotor regions important for cognitive, motor, and sensory processes. In another intervention study, Merriman et al. found that cognitive training focused on spatial navigation and obstacle avoidance training with balance control was successful in improving egocentric spatial processing and executive function in older adults. In terms of pathological aging, Li X. et al. compared older adults with and without cerebral microbleeds and found that cerebral microbleeds were closely associated with worse cognitive and motor performance on dual-task performance-based tests. Beauchet et al. reveal an overlap between late-life depressive symptomatology and motoric cognitive risk syndrome (MCR) suggesting a complex interplay between depressive symptoms and MCR that could prove useful for prevention of dementia.

Lastly, we include a series of review studies that highlight interactions between sensory, cognitive, and motor processes. Basharat et al. and Gray et al. report on sensory interactions and links to cognition in aging. In a two-pronged review, Basharat et al. sought to investigate the rigor with which researchers studying audiovisual sensory integration account for age-abnormal declines in sensory acuity and cognitive decline. Gray et al. aimed to contribute to the field of cognitive aging by proposing a framework that details the impact of musical training on speech perception. Lastly, Bai et al. review a series of neuroimaging and electrophysiological studies that highlight both visuospatial and sensorimotor functions of the posterior parietal cortex in drawing tasks.

In summary, this unique collection expands knowledge regarding the associations among cognitive, motor, and sensory processing in aging and the implications for everyday function and application. We believe that further investigations aimed at unraveling the interwoven neural circuitry of cognitive, motor and (multi)sensory functioning in aging will promote future lines of research that will potentially guide the development of novel prognostic tools, environmental adaptations, and novel technologies, and/or aid in the development of new research-driven therapeutic interventions giving rise to solutions aimed at improving the quality of life of older adults.

## Author contributions

All authors listed have made a substantial, direct, and intellectual contribution to the work and approved it for publication.

## Funding

This work was supported by the National Institute on Aging at the National Institute of Health (K01AG049813 to JRM). Additional funding was supported by the Resnick Gerontology Center of the Albert Einstein College of Medicine. UM gratefully acknowledges funding from the European Union's Horizon 2020 Research and Innovation Program under grant agreement no. 952401 (TwinBrain—TWINning the BRAIN with machine learning for neuro-muscular efficiency). JC is supported by a Canada Research Chair (II) in Multisensory Integration and Aging.

## Conflict of interest

Author JRM has a financial interest in JET Worldwide Enterprises Inc., a digital health startup spun out of research conducted at Albert Einstein College of Medicine. The remaining authors declare that the research was conducted in the absence of any commercial or financial relationships that could be construed as a potential conflict of interest.

## Publisher's note

All claims expressed in this article are solely those of the authors and do not necessarily represent those of their affiliated organizations, or those of the publisher, the editors and the reviewers. Any product that may be evaluated in this article, or claim that may be made by its manufacturer, is not guaranteed or endorsed by the publisher.
